# 483. Use of Terbinafine in Combination with Other Systemic Anti-fungal Agents As Treatment for Invasive Mold Infections. Experience from A National Cancer Institute Designated Cancer Center.

**DOI:** 10.1093/ofid/ofac492.541

**Published:** 2022-12-15

**Authors:** Eloho Ajayi, Anna Sikora, Olga Klinkova, Lisa Cozzini, Yanina Pasikhova, Rod Quilitz, Austin R Morrison, Aliyah Baluch

**Affiliations:** Moffitt Cancer Center, Tampa, Florida; University of South Florida, Tampa, Florida; Moffitt Cancer Center, Tampa, Florida; Moffitt Cancer Center, Tampa, Florida; Moffitt Cancer Center, Tampa, Florida; Moffitt Cancer Center, Tampa, Florida; Moffitt Cancer Center, Tampa, Florida; Moffitt Cancer Center, Tampa, Florida

## Abstract

**Background:**

The severely Immunocompromised are at an increased risk of fatal infection caused by invasive molds. Amphotericin B and triazole agents with anti-mold activity are commonly used in treating invasive mold infections. There is some data from in-vitro experiments to suggest an enhanced killing of some molds when terbinafine is used in combination with certain azoles or amphotericin B. At our center, terbinafine is occasionally used in combination with other systemic anti-fungal agents in treating severely immunocompromised patients with invasive mold infections.

**Methods:**

The study was a retrospective review of electronic medical records. Sixty-four patients treated for culture confirmed infection with mold isolates at the Moffitt Cancer Center from 1/1/2015 to 6/1/2021 were included in this study. There were 2 treatment groups: Group A comprised of those treated with a terbinafine containing regimen and Group B comprised of patients that were not treated with a terbinafine containing regimen.

**Results:**

Treatment group A: Comprised of 14 patients. Twenty-nine percent (4/14) had skin/soft tissue infections, 21% (3/14) had rhinosinusitis, 21% (3/14) had pulmonary infections and 4 patients (29%) had disseminated infection. Thirty-six percent (5/14) were discharged with treatment response, 57% (8/14) did not respond to treatment or died due to the invasive mold infection and 7% (1/14) died as a result of malignancy.

Treatment group B: Had 50 patients. thirty-eight percent (19/50) had pulmonary infections, 28% (14/50) with skin/soft tissue infections, 16% (8/50) had rhinosinusitis, 2% (1/50) were diagnosed with central nervous system infection, 2% (1/50) had gastrointestinal infection, 14% (7/50) had an unclear source of infection. Forty-eight percent (24/50) were discharged with treatment response, 42% (21/50) had treatment failure or died due to the invasive mold infection and 10% (5/50) died as a result of malignancy.

**Table 1:**
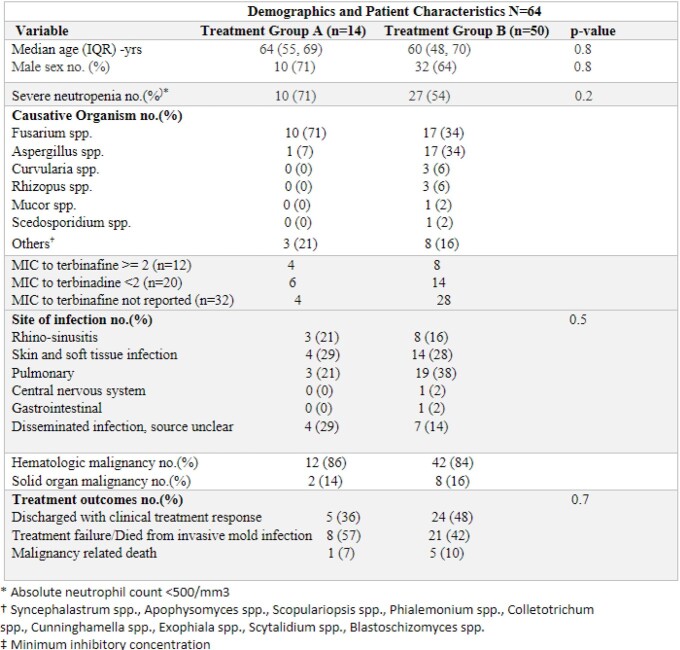

**Figure 1:**
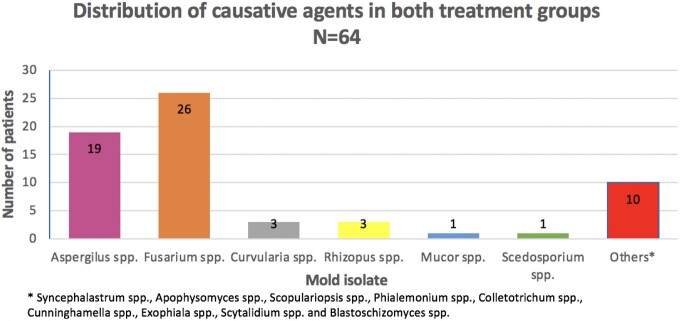

**Conclusion:**

There was no statistically significant improvement in outcome observed in this study when terbinafine was used in addition to other systemic anti-fungal medications as treatment of invasive mold infections. There is a need for larger studies to determine if terbinafine has a role in the treatment of invasive mold infections.

**Disclosures:**

**All Authors**: No reported disclosures.

